# Identification of Warning Transition Points from Hepatitis B to Hepatocellular Carcinoma Based on Mutation Accumulation for the Early Diagnosis and Potential Drug Treatment of HBV-HCC

**DOI:** 10.1155/2022/3472179

**Published:** 2022-09-05

**Authors:** Fei Xu, Qingkang Meng, Feng Wu, Yakun Wang, Wenjun Yang, Yun Tong, Lei Liu, Xiujie Chen

**Affiliations:** ^1^Department of Pharmacogenomics, College of Bioinformatics Science and Technology, Harbin Medical University, Harbin 150081, China; ^2^Department of Orthopedic Surgery, The 2nd Affiliated Hospital of Harbin Medical University, Harbin 150001, China

## Abstract

The accumulation of multiple genetic mutations is essential during the occurrence and development of hepatocellular carcinoma induced by hepatitis B (HBV-HCC), but understanding their cooperative effects and identifying the warning transition point from hepatitis B to HCC are challenges. In the genomic analysis of somatic mutations of the patient with HBV-HCC in a patient-specific protein-protein interaction (ps-PPI) network, we find mutation influence can propagate along the ps-PPI network. Therefore, in the article, we got the mutation cluster as a new research unit using the Random Walks with Restarts algorithm that is used to describe the efficient boundary of mutation influences. The connection of mutation cluster leads to dysregulation of signaling pathways corresponding to HCC, while dysregulated signaling pathways accumulate gradually and experience a process from quantitative to qualitative changes including a critical mutation cluster called transition point (TP) from hepatitis B to HCC. Moreover, two subtypes of HCC patients with different prognosis and their corresponding biological and clinical characteristics were identified according to TP. The poor prognosis HCC subtype was associated with significant metabolic pathway dysregulation and lower immune cell infiltration, while we also identified several preventive drugs to block the transformation of hepatitis B to hepatocellular carcinoma. The network-level study integrated multiomics data not only showed the sequence of multiple somatic mutations and their cooperative effect but also identified the warning transition point in HCC tumorigenesis for each patient. Our study provides new insight into exploring the cooperative molecular mechanism of chronic inflammatory malignancy in the liver and lays the foundation for the development of new approaches for early prediction and diagnosis of hepatocellular carcinoma and personalized targeted therapy.

## 1. Introduction

Hepatocellular carcinoma (HCC) is a leading cause of cancer-related deaths worldwide, with a 5-year survival rate of 18% [1]. Hepatitis B virus (HBV) infection is a major risk factor for HCC tumorigenesis [2]. Although several studies have described the molecular mechanisms that hepatitis B drives hepatocellular carcinoma (HBV-HCC) and proposed some therapeutic strategies [3–6], the identification of transition point for early diagnosis of inflammatory-to-cancer transformation in HBV-HCC is an open question.

HCC tumorigenesis is usually accompanied by the accumulation of somatic mutations; these somatic mutations regulate different signaling pathways such as cell cycle, signal transduction, and stress response [7, 8], and different signaling pathway dysfunctions frequently occur simultaneously in cancer [9], so we can infer that these signaling pathway dysfunctions provide an advantage to the growth of cancer cells. Changing analysis of these signaling pathways from quantitative to qualitative views supplies an insight into HCC tumorigenesis, which should be essential in evaluating the transition point of tumorigenesis for the early diagnosis of HCC. To investigate the transformation mechanism of hepatitis B to HCC, Chen et al. identified dynamic network biomarkers to detect the critical states (meaning the transition point) of many disease progression using nonlinear dynamic theory [10], and Liu et al. explored a novel landscape dynamic network biomarker methodology to identify early-warning signals of complex diseases on a single-sample basis [11]. These efforts have provided new ideas for the early detection of tumors and focus more on gene expression rather than the biological processes regulated by genes; however, the biological processes regulated by genes better represent the evolution of tumors. To understand the transformation mechanism of hepatitis B driving HCC, our study researched the transition points from quantitative changes to qualitative changes during the accumulation of somatic mutations and their associated signaling pathways in a complex PPI network of HCC patients; this overcomes the bias of individual gene expression. Our work not only describes the synergistic effects of mutated genes during uncontrollable inflammatory progression but also demonstrates the interplay of signaling pathways during tumor evolution.

Studies have shown that the progression of many complex diseases has an unstable predisease state, which can be reversed back to a normal state if treated appropriately [12]. Here, to further explore the unstable predisease state of HBV-HCC, the percolation theory is used to simulate the process of tumorigenesis. The percolation theory is a physical concept describing random propagation and flow of fluid in random porous disordered media and there is a sudden transition point with the change of the random plugging degree of the pores, which is often called percolation transition [13]. We used the PPI network as the random porous disordered media, the somatic mutations as the events of blocked pores, and a sudden transition point as the percolation transition of tumorigenesis.

In our study, we used a mutation cluster as the basic research unit to identify each patient's transition point (TP) rather than single dominance of somatic mutations, which not only reflected the synergism between these genes but was also more consistent with the HCC tumorigenesis. We constructed patient-specific protein-protein interaction (ps-PPI) networks and extracted more representative largest connected clusters (LCCs). And then, we calculated the HCC propensity score (HCC-PS) of genes in LCCs, while HCC-PS included the biological functions of genes, considered the cooperation mechanism between genes, and found the TP of each patient from hepatitis B to HCC. Finally, we proposed a novel subtype model based on the TPs; the patients in subtype 1 had metabolic dysregulation and low immune levels and had a worse prognosis. Our finding is crucial for early detection, prevention, and treatment of HBV-HCC.

## 2. Material and Methods

### 2.1. Obtaining and Processing Data

The information of 89 stage I HBV-HCC patients in China (CHCC-HBV) was obtained from the published research, including gene expression profile and proteomic data, somatic mutation profile, and clinical profile of patients with HCC and hepatitis B [14]. 68 advanced-stage patients in CHCC-HBV and 102 HBV-HCC patients in TCGA were selected for validation [14].

The human protein-protein interaction relationships with a combined score greater than 700 in the STRING database were obtained [15], which maximizes patient information retention while maintaining high confidence in protein-protein interactions (nodes = 14805, edges = 361152). 218 HCC-associated driver genes were obtained from the DriverDBv3 [16]. There are 529 pathways, including 14851 genes in the Kyoto Encyclopedia of Genes and Genomes (KEGG) database [17]. We downloaded information on 519 pathways, including 28 cancer-related pathways and 1 hepatocellular carcinoma-related pathway. The CERES scores of genome-scale CRISPR-Cas9 knockout for 18333 genes in 20 HCC cell lines were acquired from the Dependency Map (DepMap) portal [18]. CERES score is used to measure the dependency of the mutations in cancer cell lines (CCLs), and a lower score indicates that the gene is more essential in cell growth and survival of a given CCL. Differentially expressed genes (DEGs) between hepatitis B and HCC were identified using the R package “limma” [19]; adj *p* value < 0.05 and |log2FC| > 1 were set as the threshold. Combined with gene expression profiles and clinical profiles, the univariate Cox proportional hazard regression model was used to screen overall survival- (OS-) associated genes (*p* value < 0.05). For each patient, the protein-protein interactions in which two gene expression values were greater than 0 in hepatitis B and HCC were extracted and the maximal interconnected network was defined as ps-PPI.

### 2.2. Mining Mutation Cluster and Connected Clusters (CC) in ps-PPI

It has been found that genes causing the same or similar diseases are usually close to each other in PPI [20]. Set genes with mutations as seeds separately to model their influence over the nonmutated neighbor genes in each ps-PPI using the Random Walks with Restarts (RWR) algorithm [21]. According to the RWR algorithm, the score of each nonmutated neighbor gene around the mutated gene was calculated according to the following formula:

(1) $$\begin{array}{c} P_{t+1}=\left(1-r\right)MP_{t}+rP_{0}. \end{array}$$

In the formula, *r* is the probability of one gene transferring to its neighboring gene, while the probability of one gene transferring to itself is 1 − *r*. According to previous studies, *r* = 0.7 is suitable for RWR algorithm [22, 23]. The vector *P*_0_ is a binary matrix (0, 1), 0 indicates a nonmutated gene and 1 indicates a mutated gene. *M* is the adjacency matrix of ps-PPI, and the mutated gene seeks its affected neighborhood along with *M*. *P*_*t*_ eventually converges, and *P*_*t*+1_ reaches a steady state when the value of *P*_*t*_ and *P*_*t*+1_ difference is less than 1*e* − 10 in this study [23–25].

X-cluster was a series of genes centered on a gene X with mutation and spread the influence of X over the nonmutated neighbor genes in the ps-PPI. The closer the nonmutated neighbor gene was to X, the higher the score was. A series of mutation clusters are scattered in the ps-PPI and connected by nonmutated genes; therefore, the connected cluster (CC) is some connected mutation clusters in the ps-PPI.

### 2.3. Exploring the Order of Mutation Clusters in the Largest Connected Cluster (LCC)

Each mutation cluster is a small subnet, while each connected cluster is a bigger subnet; the largest connected cluster (LCC) is the biggest subnet in the ps-PPI that is formed by some connecting CCs. This study is based on the idea of continuous somatic mutation during carcinogenesis. Shin et al. proposed the mutation-select-rule to choose the next mutation cluster, minimizing the size of the subnet on the premise that there was overlap among the previous mutation clusters [26]. We simulated the sequence of clusters during LCC formation. In each patient, a cluster centered on passenger mutation was first selected as the start, and the next cluster was added according to the mutation-select-rule until all clusters were in the LCC. Then, repeating the above steps to each cluster centered on passenger mutation, sequences of clusters were obtained with an equal number of passenger mutations. The order of any two clusters in all sequences was counted, and each patient got a unique sequence of clusters during the formation of LCC eventually.

To test the synergistic effect among the clusters in the patient's LCC to expand the effective boundary of mutation in the ps-PPI, we selected test genes randomly equal to the number of mutations in each ps-PPI, and the clusters of test genes were calculated and concatenated into LCC (test) and repeated 500 times. Finally, we compared the size of LCC (test) and that of intrinsic mutations.

### 2.4. Identify the Transition Point (TP) of Each Patient

The unique sequence of clusters during the formation of LCC was obtained for each patient, and most passenger mutation clusters have no obvious oncogenic effect. However, the accumulation of these passenger mutation clusters with one or several driver mutation clusters will alter the biological process and induce cancer. According to the sequence of clusters, the HCC propensity score (HCC-PS) was calculated for each cluster joined in the subnet to form the LCC. The HCC-PS was completely specific by using KEGG pathway information and each patient's gene expression profile in subnet according to the following equation. HCC-PS value represented the likelihood that the patients transform from hepatitis B to HCC, and the cluster with the largest HCC-PS was selected as the TP from hepatitis B to HCC.

(2) $$\begin{array}{c} \text{HCC}‐\text{PS}=\overset{p}{\underset{1}{\sum }}P_{i}. \end{array}$$

In the equation, *p* is the number of KEGG pathways in which genes of subnet enriched in *p* < 0.01, and *P*_*i*_ is the propensity score of pathway *i*. The formula of *P*_*i*_ is as follows:

(3) $$\begin{array}{c} P_{i}=G\overset{g}{\underset{j=1}{\sum }}W\times R_{j}, \end{array}$$

(4) $$\begin{array}{c} G=\frac{G_{h}}{G_{i}}, \end{array}$$

(5) $$\begin{array}{c} W=\frac{w_{j}}{W_{j}}. \end{array}$$

Here, *g* denotes the number of genes in the subnet that are enriched in pathway *i*. *G* is the correlation coefficient between pathway *i* and the HCC pathway. *W* is the oncogenicity coefficient of gene *j*. *R*_*j*_ is the difference in gene *j* expression between HCC and hepatitis B. *G*_*h*_ is the number of intersection genes in pathway *i* with genes in the HCC pathway, and *G*_*i*_ is the number of genes in pathway *i*. *w*_*j*_ is the number of cancer pathways containing gene *j*, and *W*_*j*_ is the number of KEGG pathways containing gene *j*.

### 2.5. Selection of Candidate Genes and Establishing Subtype Model

Patients were classified into two subtypes according to the TPs, including subtype 1 with TP53 cluster as a TP and subtype 2 with no fixed biomarker cluster as TP. The subtype model was constructed in two steps. Firstly, subtype-related genes were the intersection of the following three datasets: (1) DEGs of HCC in two subtypes (adj *p* value < 0.05, |log2FC| > 1); (2) DEGs of hepatitis B in two subtypes (adj *p* value < 0.05, |log2FC| > 1); and (3) receiver operating characteristic (ROC) curves were plotted using the R package “pROC,” and the area under the curve (AUC) was calculated to assess the accuracy of the model [27]; the ROC curves of two subtypes in HCC and hepatitis B were plotted, respectively, the genes with both AUC greater than 0.6. Secondly, the least absolute shrinkage and selection operator (LASSO) logistic regression model was used to extract the feature genes and their coefficient from subtype-related genes. The formula of the subtype model is as follows, and *i* is feature genes:

(6) $$\begin{array}{c} \text{subtype model}=\underset{\text{i}}{\sum }\text{c}\text{oefficient }\left({\text{gene}}_{i}\right)*\text{expression }\left(\;{\text{gene}}_{i}\right). \end{array}$$

To validate the accuracy of the subtype model, 68 advanced-stage patients from CHCC-HBV and 102 HBV-HCC patients in TCGA were used. The TPs were identified for each patient according to the HCC-PS, and the AUC was calculated by plotting the ROC curve based on the subtype model.

### 2.6. Functional Enrichment Analysis Based on Multiomics Data

The differences in biological processes between hepatitis B and HCC in subtype 1 patients were analyzed. The “c2.cp.kegg.v7.4.entrez.gmt” gene set was downloaded from the MSigDB database [28]. And the R package “clusterProfiler” [29] was used for gene set enrichment analysis (GSEA) (adj *p* value < 0.01). Gene set variance analysis (GSVA) was performed using the R package “GSVA” [30]. The difference in the pathway between hepatitis B and HCC in subtype 1 patients was identified by “limma” algorithm (adj *p* value < 0.01). The results of transcriptome-based GSEA and proteome-based GSVA were intersected to obtain the differential pathways between hepatitis B and HCC.

Single-sample gene set enrichment analysis (ssGSEA) was implemented to analyze the enrichment level of 29 immune signatures via invoking the R package “GSVA” [31, 32], combined with the “limma” for differential immune signatures between hepatitis B and HCC in the patients of subtype 1 (adj *p* value < 0.01).

### 2.7. Core Target Screening and Drug Identification

TP is defined as the threshold for the transformation from hepatitis B to HCC. The genes before TP during the formation of LCC can be used as drug targets to block cancer progression. A three-step analysis was performed to find candidate drug targets in subtype 1 patients. Firstly, DEGs between hepatitis B and HCC in subtype 1 patients were selected (adj *p* value < 0.05, |log2FC| > 1). DEGs with AUC > 0.9 were used to construct a transformation model from hepatitis B to HCC. Secondly, the genes before TP were identified for 51 patients separately, and the shared genes were defined as the intersection of the above 51 gene sets. The Pearson correlation coefficients (PCC) of shared genes expression with the transformation model were calculated (|*r*| > 0.5, *p* < 0.05). In the third step, the PCC of the CERES score and transformation model in HCC cell lines were calculated to screen for the poor-prognosis genes associated with HCC (|*r*| > 0.5, *p* < 0.05). The approved drugs of available drug targets in subtype 1 patients were obtained from the DrugBank database [33].

We screened 16 HCC cell lines with 95 compounds, and the drug response data and genomic markers of sensitivity were obtained from the Genomics of Drug Sensitivity in Cancer database [34], and the R package “oncoPredict” [35] was used to predict drug sensitivity of HCC patients in subtype 1.

## 3. Results

### 3.1. Unique ps-PPI

**Figure 1:**
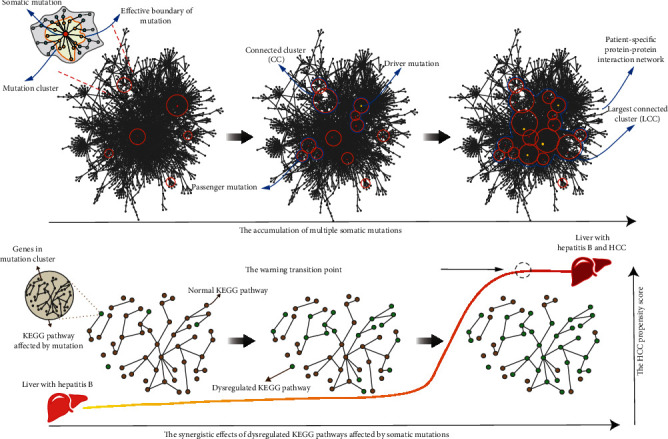
The accumulation of multiple somatic mutations and the synergistic effects of dysregulated pathway affected by somatic mutation in the HCC tumorigenesis.

To explore the timing of transition point (TP) onset more accurately, we only chose phase I HCC patients. To simulate the accumulation of multiple somatic mutations and the synergistic effects of dysregulated pathways affected by somatic mutation during the HCC tumorigenesis, we constructed a ps-PPI for each HCC patient in this study (Figure 1). The number of interactions in each ps-PPI was counted (maximum 360830 and minimum 355666), and the number of genes in ps-PPIs was similar (minimum 14742 and maximum 14801), but the genes are different. Patients had a wide range of mutated genes from 30 to 610 and a few driver mutations from 1 to 20, while the ps-PPI preserved at least 80% of the mutated genes for each patient.

In short, the number of genes and interactions in ps-PPIs was similar; however, mutated genes and gene expression values in each patient differed significantly. So ps-PPIs were high specificity and reflected the necessity of personalized treatment.

### 3.2. The Mutation Clusters as Research Units

In the ps-PPIs, we found that the genes with mutation scattered in various positions and were closely related to the nonmutated genes. We set the mutation cluster as a research unit, which consisted of a gene with mutation and its nonmutated neighbors. RWR algorithm was used to find the mutation cluster in the ps-PPIs; a score threshold of 0.001 was set to ensure a high correlation between genes in each mutation cluster while keeping the number of genes in each mutation cluster from being 0. The average size of the mutation cluster was 324, with the largest mutation cluster having 1095 genes and the smallest mutation cluster having 2 genes; it can be seen that the influence of different mutations on the network varies widely. At the same time, there are connections between clusters to form CCs in the ps-PPIs, and the largest CC is called LCC (Figure 1).

**Figure 2:**
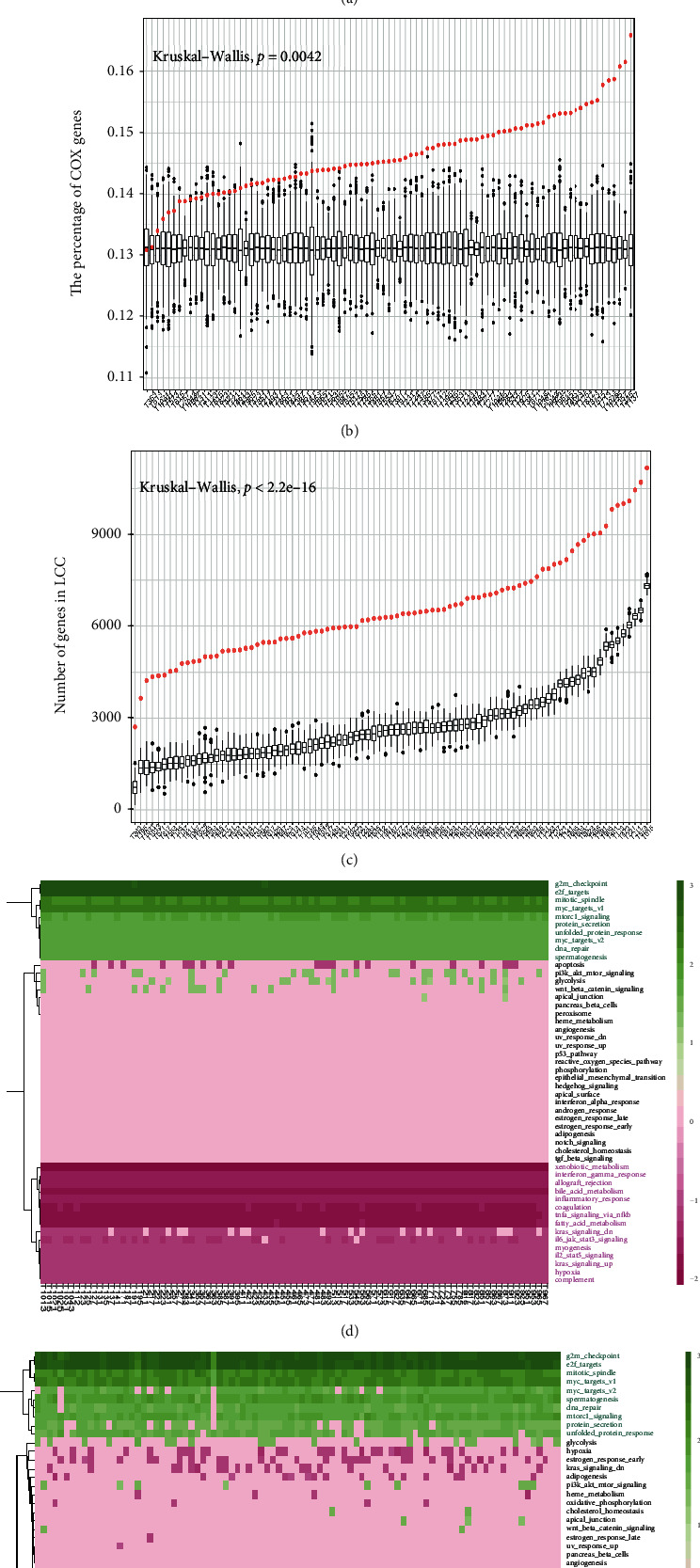
Analysis of LCCs. (a, b) The proportion of DEG/OS-associated genes in LCCs vs. that in LCCs (test), red dots represent LCCs and black dots represent LCCs (test). (c) The size of LCCs and the size of LCCs (test) in each patient, red dots represent LCCs and black dots represent LCCs (test). (d, e) In the biological processes of genes in LCCs and ps-PPIs, green indicates that the hallmark is activated, and pink indicates that the hallmark is suppressed.

The number of genes in each patient's LCC was determined by the overlap and synergistic effect of CCs. Although there were only 106 somatic mutations in ps-PPIs on average (1% genes of the ps-PPIs), the size of LCCs affected by somatic mutations covered 50% of the ps-PPIs. The number of driver mutations in LCCs ranged from 1 to 16, but the number of nonmutated driver genes affected by passenger mutations in LCCs ranged from 68 to 162, which means a few mutations could have a greater impact. Moreover, 3573 DEGs between HCC and hepatitis B were identified, including 1533 upregulated and 2040 downregulated. The univariate Cox proportional hazard regression model was performed, and 2275 OS-associated genes of HCC patients were identified. The proportion of DEG/OS-associated genes in LCCs showed significant differences because of strong individualization. The proportion of DEG/OS-associated genes in LCCs with that in LCCs (test) was compared; LCCs contained more DEGs (*p* = 4.8*e* − 5, Figure 2(a)) and more OS-associated genes (*p* = 0.0042, Figure 2(b)). The result showed that there was not only an overlapping effect but also a synergistic effect between CCs. The size of LCCs composed of intrinsic mutations was significantly larger than the size of LCCs (test) (*p* < 2.2*e* − 16, Figure 2(c)). Thus, the synergistic cooperation between somatic mutations in cancer cannot be attributed to random selection; it could be determined by the topological properties of somatic mutations in the ps-PPIs.

To assess whether LCCs can represent ps-PPIs, we compared the differences in the biological processes of genes involved in ps-PPIs and LCCs; the gene set “h.all.v7.4.symbols.gmt” from MSigDB database was used for GSVA. The result showed that the genes in LCCs and the genes in ps-PPIs activated the same biological processes, such as E2F targets, G2M checkpoint, mitotic spindle, and MYC target proliferation processes while suppressing the xenobiotic metabolism, allograft rejection, bile acid metabolism, and inflammatory response, which were highly correlated with HCC tumorigenesis. LCCs could represent the patient's biological function while more fully presenting personalized information (Figures 2(d) and 2(e)).

### 3.3. TP Identified for Each HCC Patient

From the above results, the formation of LCCs resulted from the collaborative effect of CCs and the genes in LCCs were closely related to the cancer-related function. Then, identifying the CC development sequence in LCCs was important for cancer prevention, early diagnosis, and treatment. According to the mutation-select-rule [26], a sequence of mutation clusters was obtained for each patient, and most driver mutations observed in HCC have been optimized to maximize the transition from hepatitis B to HCC after some passenger mutations.

**Figure 3:**
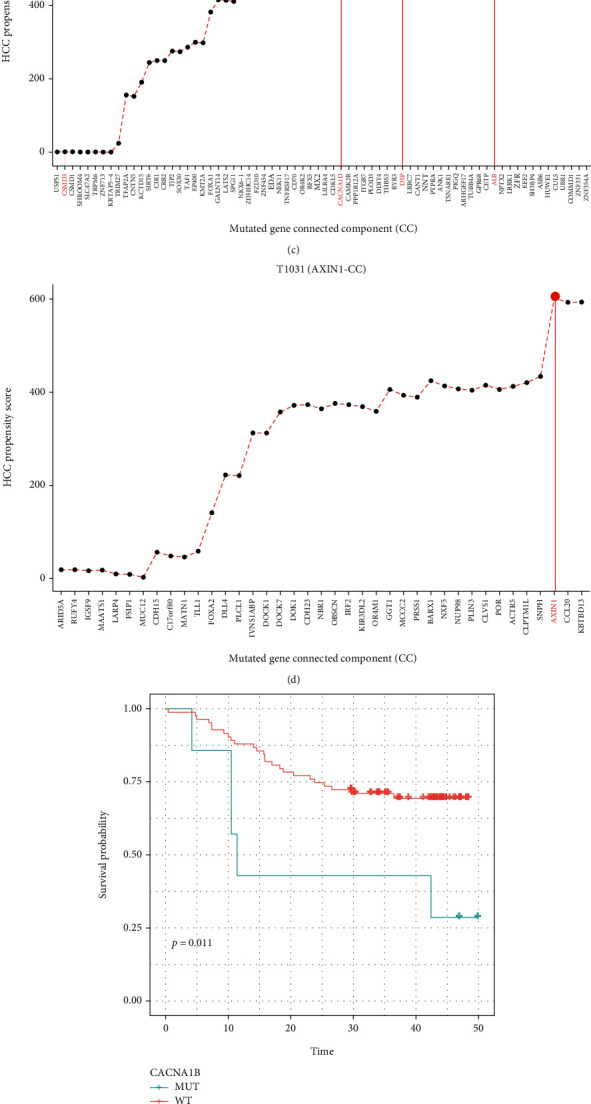
HCC-PS curve and patient subtypes. (a–d) Examples of HCC-PS curve. The horizontal axis is the order of mutations which means the cluster sequence, and the vertical axis is the HCC-PS. The red indicates the driver gene. (e) Kaplan-Meier plot showing different prognoses of CACNA1B-mutated patients vs. CACNA1B-WT patients. (f, g) Kaplan-Meier plot showing different prognoses of subtype 1 vs. subtype 2 in stage I CHCC-HBV patients and advanced-stage CHCC-HBV patients.

To identify the TP in the transformation from hepatitis B to HCC, the biological function of genes in the subnet at the time cluster joined in following the sequence of the clusters was assessed according to the HCC-PS, and the HCC-PS curve was plotted (Figures 3(a)–3(d)). The results showed that the HCC-PS increased slowly with the addition of the clusters of the passenger mutations; the HCC-PS underwent a sudden increase abruptly when a cluster of the driver mutation was added. And after that, the HCC-PS reverted to steady again until the next driver mutation and its CC were added. The HCC-PS reached the highest point, which was defined as TP, and the patient completed the transformation from hepatitis B to HCC.

Taking patient T923 as an example (Figure 3(a)), the patient has 55 genes with mutation, including 8 driver mutations. The LCC of patient T923 consisting of 5593 genes was obtained from ps-PPI; there were 53 mutations including 8 driver mutations (8 driver mutations were ARID1A, CACNA1B, CPS, HNF1A, CUBN, FGA, ZNF451, and TP53). The 53 HCC-PSs were calculated during the gradual joining of the 53 mutation clusters. It was found that the clusters of driver mutations ARID1A, HNF1A, and TP53 led to HCC-PS dramatic increase; the clusters of passenger mutations kept the HCC-PS stable due to the homeostasis. ARID1A was an important tumor suppressor gene, and it played a key role in the proliferation, differentiation, and apoptosis processes [36]. CACNA1B mutation type led to a lower survival rate compared with wild type in HBV-HCC patients (*p* < 0.05, Figure 3(e)). HNF1A was a novel oncogene that regulates the stem cell properties of human pancreatic cancer [37], and the key role of HNF1A in HCC was yet to be discovered. Under the synergistic influence of preceding mutations and their clusters, the HCC-PS of the subnet became progressively larger and reached its maximum until the TP53 cluster joined into the LCC, and the patient completed the transformation from hepatitis B to HCC.

The TPs of patients were counted; the TP53 cluster appeared as the TP in 51 patients, while 38 patients had other driver mutations as TPs, including AXIN1 cluster, CTNNB1 cluster, and other low-frequency genes. Therefore, patients were divided into two subtypes, subtype 1 composed of 51 patients with TP53 cluster as TPs and subtype 2 of 38 patients with no fixed gene as TPs. The survival time of patients in subtype 1 was significantly shorter (*p* = 0.019, Figure 3(f)), and the patients with advanced stages of CHCC-HBV in subtype 1 had a shorter survival time than patients in subtype 2 (*p* = 0.023, Figure 3(g)).

### 3.4. Validation of Patient Subtype Model

**Figure 4:**
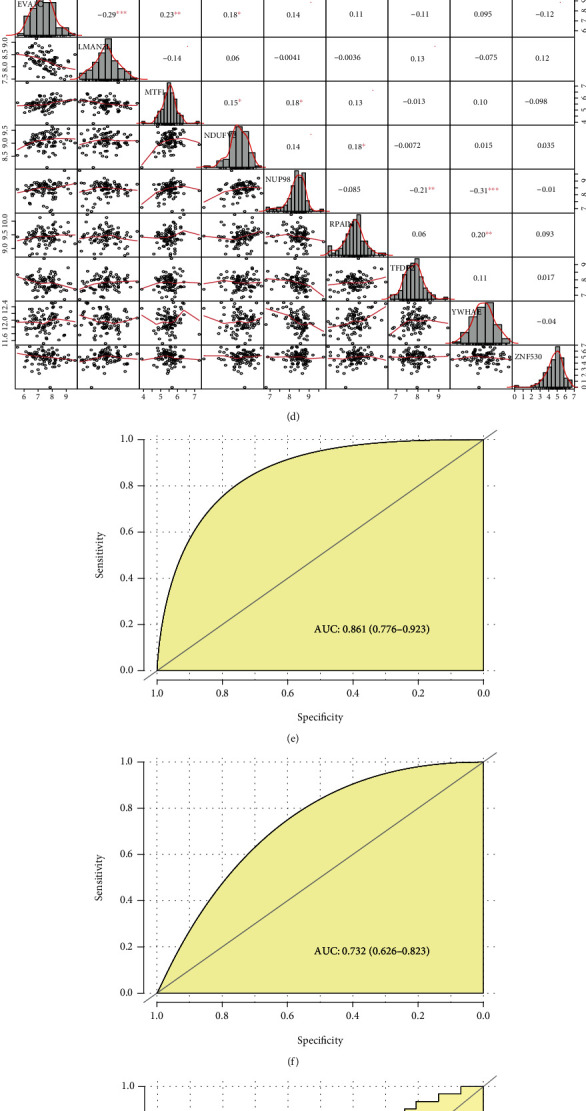
Construction and validation of patient subtype model. (a) Venn diagram of subtype-associated genes. (b) Semantic similarity of subtype-associated genes. (c, d) The PCCs for gene expression levels of the 9 signature genes in HCC and hepatitis B. (e, f) The ROC curve of patient subtype model in HCC and hepatitis B. The ROC curve of the subtype model in HCC of CHCC-HBV patients with advanced stages (g), hepatitis B of CHCC-HBV patients with advanced stages (h), and HCC of TCGA-HBV patients (i).

A three-step analysis was performed, 39 subtype-related genes were selected in subtype 1 patients (Figure 4(a)), and 9 signature genes and their coefficients were obtained using the LASSO regression (EVA1C: 0.065, LMAN2L: 0.184, MTF1: 0.051, NDUFV3: 0.237, NUP98: 0.054, RPAIN: 0.168, TFDP2: -0.067, YWHAE: 0.183, and ZNF530: -0.122). The semantic similarity among GO terms between 9 genes was less than 0.5 calculated by the R package “GOSemSim” [38] (Figure 4(b)). The PCCs for gene expression levels of the 9 signature genes also showed a weak correlation in HCC and hepatitis B; the highest PCC was 0.48 in HCC and 0.31 in hepatitis B separately (Figures 4(c) and 4(d)); the results showed that 9 signature genes had a wide range of biological function.

The subtype model showed an AUC of 0.861 in HCC (Figure 4(e)) and 0.732 in hepatitis B (Figure 4(f)). So, the patient subtype model showed good classification efficacy in both HCC and hepatitis B. Validation of the subtype model using the CHCC-HBV patients with advanced stages showed an AUC value of 0.650 in HCC (Figure 4(g)) and 0.700 in hepatitis B (Figure 4(h)). The subtype model was also further validated in TCGA dataset with an AUC of 0.799 (Figure 4(i)). The above results indicated that the subtype model could well identify the patient with TP53 cluster as the TP.

### 3.5. Characteristic Changes between Hepatitis B and HCC in Subtype 1 Patients

**Figure 5:**
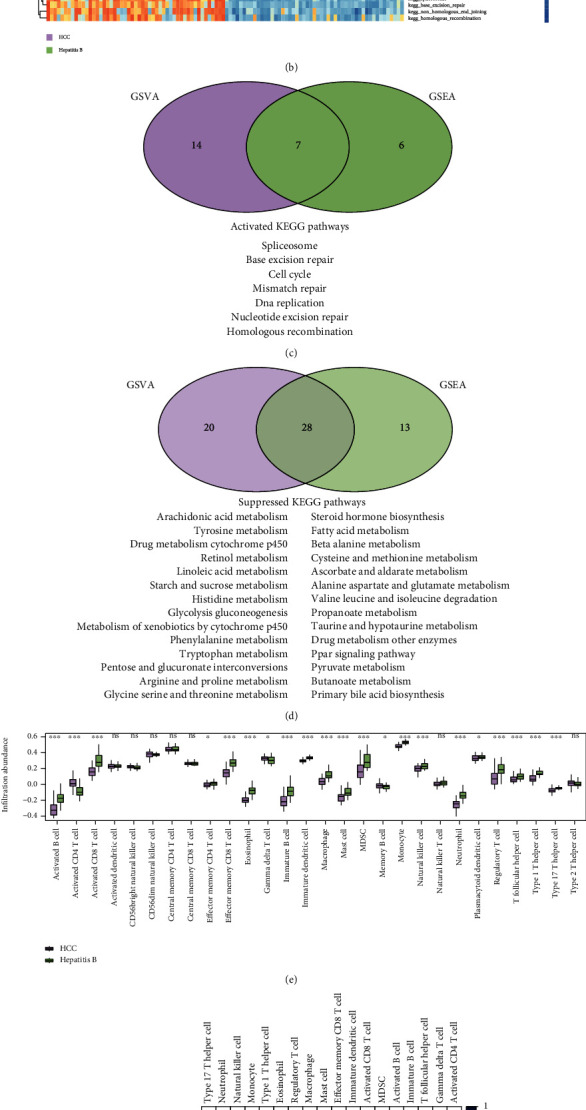
Enrichment analysis and immune infiltration analysis in subtype 1 patients. (a) Transcriptome-based GSEA, node size indicates the number of genes in the current pathway and node color indicates the significance of the results. (b) Proteome-based GSVA, purple represents HCC and green represents hepatitis B, red indicates that the pathway is activated, and blue indicates that it is suppressed. The Venn plot of (c) activated and (d) suppressed KEGG pathways in GSEA and GSVA. (e) Immune cell infiltration in HCC and hepatitis B, green indicates hepatitis B and purple indicates HCC. ∗ is *p* value < 0.05, ∗∗ is *p* value < 0.01, and ∗∗∗ is *p* value < 0.001. Correlation analysis of immune cells in (f) HCC and (g) hepatitis B.

To investigate the biological processes altered between hepatitis B and HCC in subtype 1 patients, we conducted transcriptome-based GSEA and proteome-based GSVA. The GSEA results showed that 21 KEGG pathways were activated and 48 KEGG pathways were suppressed in HCC (Figure 5(a)). The GSVA results showed that 13 KEGG pathways were promoted and 41 KEGG pathways were inhibited in HCC compared to HBV (Figure 5(b)). It is consistent with previous studies that cell-cycle, DNA damage repair pathways are activated in GSEA and GSVA results (Figure 5(c)) [14]. HBV infection can produce immune-mediated inflammation, which causes DNA damage in hepatocytes, and the DNA of HBV can randomly integrate into the chromosomal DNA of hepatocytes to further cause DNA damage, which plays a role in the development of HCC [39]. Taking the intersection of GSEA result and GSVA result yielded 28 metabolism-related and immunity-related KEGG pathways that were suppressed (Figure 5(d)). Some studies have shown that metabolism and immunity-related pathways play an important role in the development of HCC [40]. This suggests that patients in subtype 1 with HBV-HCC are more likely to have DNA damage as well as metabolic disturbances.

Emerging immunotherapeutic approaches are helpful in the treatment of HCC [41]. In this study, immune cell infiltration was analyzed in subtype 1 patients. 21 immune cells had a lower infiltration score in HCC (*p* < 0.01, Figure 5(e)). It was suggested that the HCC of subtype 1 was a “cold tumor,” and the effect of single immunotherapy might not be significant [42]. The correlation between immune cells in HCC was significantly weaker than that in hepatitis B (*p* < 0.05, Figures 5(f) and 5(g)). The result revealed that combined treatment was essential for HCC patients in subtype 1.

### 3.6. Potential Drugs for the Patients of Subtype 1

**Figure 6:**
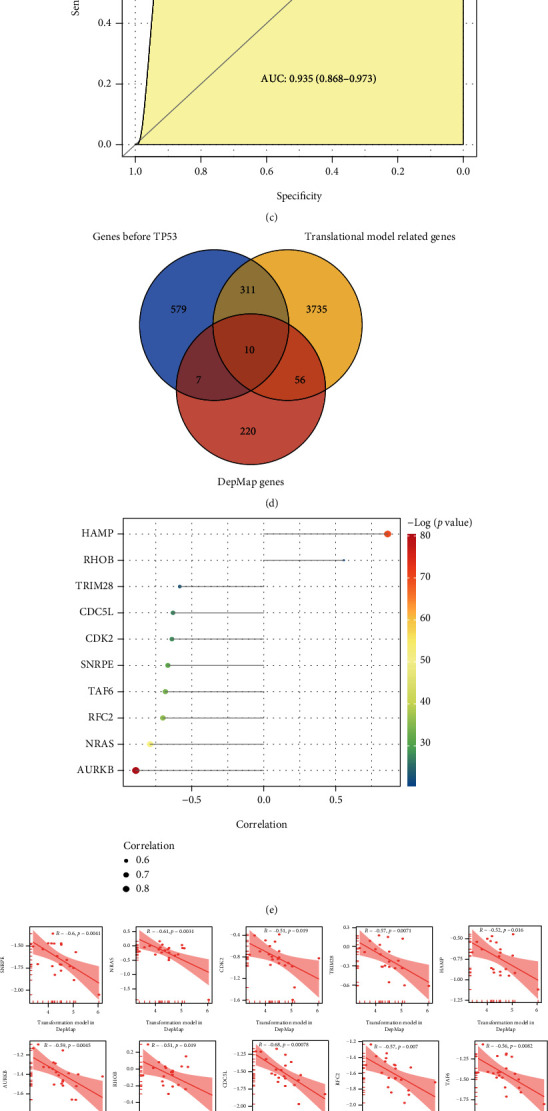
Identification of potential drugs in subtype 1 patients. (a–c) The ROC curve of the transformation model in the training set of stage I patients in CHCC-HBV, advanced-stage patients in CHCC-HBV, and patients in TCGA-LIHC. (d) Venn for potential drug targets. (e) The correlation between the expression level of 10 potential drug targets and the transformation model and CERES score. (f) The correlation between the CERES score of 10 potential drug targets and the transformation model. (g, h) Kaplan-Meier plot of 7 potential drug targets.

To find the warning biomarkers for early detection and potential drug to prevent the transformation of patients in subtype 1 from hepatitis B to hepatocellular carcinoma, we constructed a translational model from hepatitis B to HCC, combining the model with TP can more accurately identify precancerous states. And it had the AUC value of 0.989 in the training dataset of stage I patients in CHCC-HBV (Figure 6(a)), the AUC value of 0.858 in the test dataset of advanced-stage patients in CHCC-HBV (Figure 6(b)), and the AUC value of 0.935 in the test dataset of patients in TCGA-LIHC (Figure 6(c)). Genes that were highly correlated with the transformation model may have potential therapeutic implications for the patients in subtype 1. 4112 genes highly associated with the HBV-HCC transformation model were identified (|*r*| > 0.5, *p* < 0.05), and 293 genes whose CERES scores closely associated with the HBV-HCC transformation model were identified (*r* < −0.5, *p* < 0.05), and 907 shared genes before TP53 cluster during the formation of LCC in subtype 1 patients were identified. Finally, 10 potential drug targets were obtained by taking intersections of shared genes before TPs and transformation model-related genes, named SNRPE, AURKB, RHOT1, NRAS, CDK2, CDC5L, TRIM28, RFC2, TAF6, and HAMP (Figure 6(d)). The correlation of 10 genes with the transformation model was found to be greater than 0.5 (Figure 6(e)). The CERES scores of 10 genes were less than 0, which indicated that 10 genes were essential for the survival of HCC CCLs (Figure 6(f)). Kaplan-Meier plot showed that the gene expression levels of 7 genes were significantly associated with patient prognosis in subtype 1 patients (Figures 6(g) and 6(h)). Therefore, we think these genes can be used as potential drug targets to block the transformation from hepatitis B to HCC, inhibiting the function of these genes in subtype 1 patients may have great preventative efficiency.

Next, drugs corresponding to the potential drug targets were screened in the DrugBank database. There were 8 approved drugs for AURKB, CDK2, and HAMP. For example, hesperidin, which targeted AURKB, has the function of hepatoprotective, therapeutic drug-related liver injury, improving inflammatory response, and preventing HCC formation in rats [43, 44]; it could illustrate the accuracy of the drug targets identified in our study. The study also provided a more accurate population classification for hesperidin to prevent and treat HCC. Higher gene expression of AURKB and CDK2 led to a poorer prognosis for HCC patients. Bosutinib is an inhibitor of CDK2 for chronic granulomatous leukemia treatment [45], and fostamatinib is an inhibitor of AURKB for persistent/chronic adult immune thrombocytopenia treatment [46]. So, inhibiting the expression of AURKB and CDK2 may prolong HCC patients' survival time.

**Figure 7:**
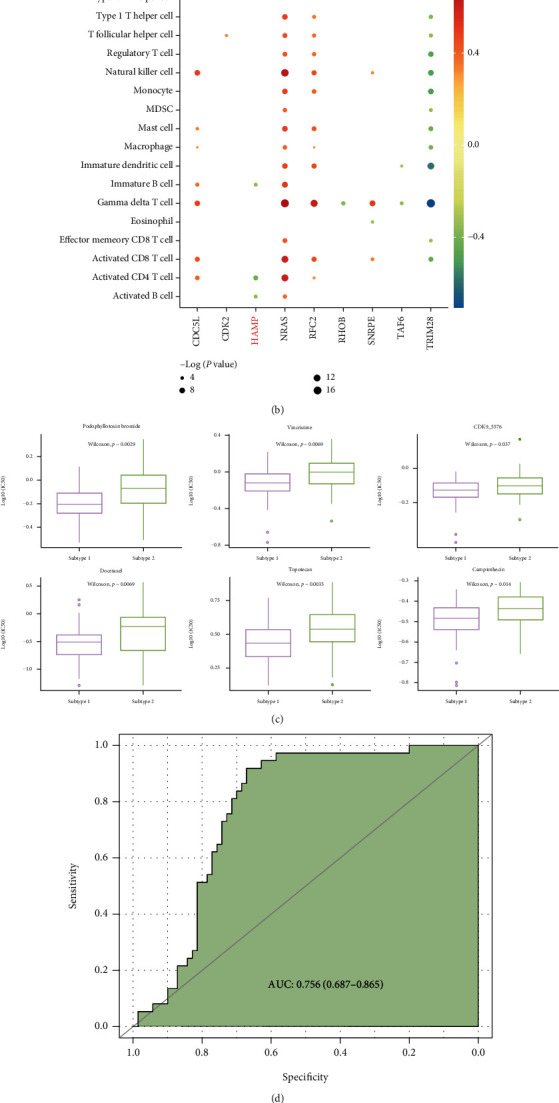
Identification of immunotherapy targets. (a, b) Correlation of 10 potential drug targets with immune cells in HCC and HBV. Node size indicates the significance, and node color indicates the PCCs. (c) Drugs for patients in subtype 1 who have completed the transformation from hepatitis B to HCC. (d, e) ROC curve of the transformation model in GSE121248.

“Cold tumors” were often associated with a poor prognosis. Targeted activation of specific kinases can promote the content of immune cells to form an inflammatory tumor environment and then convert “cold tumors” to “hot tumors,” thereby enhancing the efficacy of immune checkpoint inhibitors [47]. To analyze whether potential targeted drugs have this effect, we studied the correlation between 10 genes and immune cells. The results showed that the HAMP gene expression level in HCC was positively correlated with multiple immune cells (*p* < 0.01, Figure 7(a)), whereas it was negatively correlated with multiple immune cells in hepatitis B (*p* < 0.01, Figure 7(b)). Meanwhile, the lower expression level of HAMP leads to a poorer prognosis in HCC patients, so HAMP may be a key target for immunotherapy in HCC patients of subtype 1. This study suggests that HAMP as a kinase may have the function to activate immune cells and prevent the transformation from hepatitis B to HCC.

We identified the potential drugs for personalized prevention from hepatitis B to HCC. And for HCC patients in subtype 1 who have completed the transformation from hepatitis B to HCC, we screened more sensitive drugs for these patients compared to patients in subtype 2; the results show that podophyllotoxin, bromide, docetaxel, vincristine, topotecan, CDK9_5576, and camptothecin may have a better therapeutic effect for patients in subtype 1 (Figure 7(c)).

## 4. Discussion

The transition from normal cells to cancerous cells results from the accumulation of somatic mutations [48]. During this process, the TP can lead to the inflammatory cellular transition toward cancer cells. We used percolation theory to model this transition and propose a research flow based on the accumulation mechanism of signaling pathway affected by mutant genes in the PPI network to identify transition points and to type HCC. First, we defined a new study unit, cluster, which is centered on a mutant gene and the nonmutated gene neighbors affected by it in a patient-specific protein-protein interaction (ps-PPI) network. Then, the HCC propensity score was created to describe the dynamical properties of signaling pathway accumulation during the growing process of the ps-PPI; the HCC patients with different transition points and their corresponding biological and clinical characteristics were identified and analyzed. Finally, we proposed a novel subtype model based on the TPs; the patients in subtype 1 had metabolic dysregulation and low immune levels and had a worse prognosis. Different therapeutic strategies for hepatitis B patients and HCC patients of subtype 1 were absent. For hepatitis B patients, we found the therapeutic targets and their drugs to block the further progression from hepatitis B to HCC. And for HCC patients, we demonstrated more sensitive antioncology drugs relative to HCC patients of subtype 2.

In previous research, using data from REVEAL-HBV, 17 risk genes were developed to predict the development of HBV-HCC [49]. Nevertheless, most prognostic markers have been developed for all HCC patients without focusing on individualized management and treatment, which exhibits a deficiency in precision treatment and preemptive prevention before HCC. In this study, 89 ps-PPIs were constructed, and the unique somatic mutations and gene expression levels of each patient make the ps-PPI an accurate and highly personalized basis for subsequent studies. The ps-PPI provides more personalized medical guidance for patients with the same cancer type and stage. Our approach identified predisease states and biomarkers to diagnose and interfere with disease onset early. 10 potential drug targets and their 8 approved drugs for patients in subtype 1 were also further identified. The majority of the 8 approved drugs we identified were related to oncology treatment, demonstrating the potential for drug repositioning. These results are important for reducing the incidence and mortality of HBV-induced HCC, which provide important value for the early diagnosis and preventive treatment of HBV-HCC especially. As for patients in subtype 1 who have completed the transformation from hepatitis B to HCC, we also provide more sensitive drugs which may have a better therapeutic effect.

Mutated TP53 is one of the most common genomic alterations in human tumors. TP53 encodes p53, a transcription factor that regulates many gene expressions involved in numerous cellular processes [50]. HBV can bind and inactivate p53 in vitro [51]. And recombinant adenovirus human p53 (rAd-p53, gendicine), approved by CFDA in 2013, was used to repair the mutated P53 gene in head and neck squamous cell carcinoma (HNSCC) [52]. In this study, we found TP53 mutated and formed TP53 cluster in the precancerous state in some patients, guiding the transition from hepatitis B to HCC. The AUC of the transformation model for all HBV-HCC patients in an independent test dataset GSE121248 is 0.756 (Figure 7(d)), and the AUC for patients in subtype 1 is 0.855 (Figure 7(e)). The result shows that the translational model is more accurate in diagnosis for patients in subtype 1.

## 5. Conclusion

In conclusion, this study showed the sequence of multiple somatic mutations and their cooperative effect. And it fully identified the warning transition point in HCC tumorigenesis for each patient, which reflects the individualized analysis. However, there are a few patients who have gene expression profiles, gene mutation profiles, and clinical data of both hepatitis B and HCC at the same time. Therefore, the constructed model may not broadly represent the transformation process of HBV-HCC, but the idea and method may provide a reference for others to conduct in-depth studies. And we will collect more data to expand the sample of our study for improving the reliability.

## Data Availability

The data achieved and analyzed in the current study are available in the TCGA repository (https://portal.gdc.cancer.gov/), ICGC database (https://icgc.org/), and GEO database (https://www.ncbi.nlm.nih.gov/geo/).

## Supplementary Materials

Supplementary Materials Supplementary Table 1 and Supplementary Table 2 can be checked in the supplementary files in the submission system.
