# Supplementary material for: Identification of Warning Transition Points from Hepatitis B to Hepatocellular Carcinoma Based on Mutation Accumulation for the Early Diagnosis and Potential Drug Treatment of HBV-HCC
Source: Oxid Med Cell Longev. 2022 Sep 5;2022:3472179. doi: 10.1155/2022/3472179 (PMC9467738; doi:10.1155/2022/3472179)
Supplement: Supplementary Materials — Supplementary Table 1 and Supplementary Table 2 can be checked in the supplementary files in the submission system. [file 3472179.f1.zip › Supplementary table1-the information of ps-PPIs and LCCs.pdf]

pat_id	age	TNM	PPI	PPI_gene_	mutgene	mut.dri	seed.gene	seed.dri
T1013	33	IB	359558	14789	51	2	42	2
T1015	54	IB	358886	14778	78	7	67	7
T1021	56	IB	359496	14789	77	8	66	8
T1025	68	IB	358611	14774	62	4	53	4
T1031	52	IB	360768	14800	44	1	40	1
T1043	58	IB	360201	14795	100	5	76	5
T112	69	IB	358087	14775	100	4	75	4
T123	79	IB	359126	14775	350	17	267	15
T125	62	IB	360178	14793	106	7	89	7
T127	66	IB	358921	14765	108	8	88	8
T131	50	IB	360821	14797	187	7	151	6
T135	43	IB	360538	14793	68	5	54	5
T137	50	IB	360433	14797	55	2	46	2
T141	81	IB	360336	14790	200	11	158	9
T171	46	IB	359313	14785	109	5	94	4
T187	54	IB	360830	14800	102	5	85	5
T191	51	IB	359564	14784	89	4	72	4
T195	39	IB	360093	14792	52	4	39	4
T211	46	IA	360641	14798	400	11	323	11
T217	63	IB	360391	14790	65	2	52	2
T221	56	IB	358939	14772	70	3	55	2
T223	72	IB	359691	14782	163	7	136	6
T231	60	IB	359644	14788	68	5	61	5
T257	59	IB	359827	14785	130	4	108	4
T277	63	IB	359008	14776	117	6	86	5
T283	66	IB	360425	14799	105	9	79	8
T341	63	IB	358704	14780	75	9	64	8
T353	56	IB	358125	14768	64	7	52	6
T357	56	IB	358036	14767	70	4	57	3
T361	54	IB	359868	14788	114	5	95	4
T363	54	IB	359665	14782	30	1	25	1
T385	62	IB	359834	14788	126	6	106	6
T387	48	IB	359372	14775	132	10	110	9
T391	75	IB	358952	14781	236	12	192	11
T395	35	IB	358179	14766	102	7	84	6
T411	40	IB	358828	14774	60	9	47	7
T421	58	IB	357466	14750	176	10	140	9
T423	59	IB	358164	14761	130	5	102	4
T425	61	IB	360606	14800	71	2	54	2
T433	51	IB	360273	14795	145	8	117	8
T435	56	IB	360323	14795	96	7	84	6
T445	46	IB	358649	14777	66	7	53	6
T451	69	IB	359077	14780	309	18	261	16
T455	47	IB	359559	14776	223	11	185	10
T461	63	IB	358930	14761	111	7	90	6
T465	64	IA	358062	14763	113	4	90	2
T473	55	IA	359569	14788	359	20	287	16
T477	71	IB	360188	14794	103	6	90	6
T481	60	IB	360297	14793	106	3	84	2
T483	48	IB	359517	14780	84	8	69	8

T493	63	IB	359593	14787	115	6	90	6
T513	54	IB	356349	14749	80	5	66	4
T515	53	IB	358675	14775	60	5	48	5
T517	55	IB	359588	14787	95	3	75	3
T533	36	IB	359044	14779	58	3	45	3
T545	43	IB	359582	14778	66	4	55	3
T553	36	IB	360699	14796	55	8	47	7
T563	64	IB	358995	14772	70	6	58	5
T571	40	IB	358944	14776	60	3	45	2
T573	55	IB	359777	14788	481	19	377	16
T615	64	IB	359700	14792	610	14	485	13
T617	54	IB	359137	14776	84	3	71	3
T627	52	IB	359110	14783	103	6	86	6
T635	62	IB	360191	14800	112	5	86	4
T647	38	IA	358491	14773	67	4	60	4
T665	48	IB	359694	14785	214	9	162	8
T671	48	IB	358879	14773	78	3	62	2
T685	54	IB	355666	14742	142	5	118	5
T713	54	IB	358700	14779	76	6	69	6
T721	62	IB	360357	14792	378	12	299	10
T724	81	IB	359892	14796	91	10	74	9
T737	52	IB	357899	14769	157	4	133	4
T755	63	IB	357397	14764	149	5	126	5
T785	53	IB	359307	14786	75	7	60	5
T815	47	IB	359590	14787	84	7	76	7
T817	46	IB	359928	14788	51	1	41	1
T823	41	IA	359578	14785	266	11	206	10
T851	75	IB	359617	14785	139	8	106	7
T857	65	IB	359615	14792	120	4	105	4
T863	73	IB	359483	14791	213	10	172	9
T867	65	IB	359872	14784	241	9	181	7
T873	57	IB	359262	14778	98	5	80	3
T911	67	IB	359558	14782	66	3	56	3
T923	53	IB	360108	14791	72	10	55	8
T925	61	IB	359123	14782	108	4	93	4
T951	48	IB	359645	14789	106	4	87	4
T953	61	IB	360239	14794	143	8	116	7
T965	61	IB	359863	14793	310	9	244	8
T967	50	IB	360165	14801	69	8	51	6

pat_id: patient ID
age: patient age
TNM: patient TNM stage
PPI: the number of interaction in patient-specific PPI
mutgene: the number of patient mutated genes
mut.dri: the number of mutated driver genes
seedgene: the number of mutated genes in PPI

seed.dri: the number of mutated driver genes in PPI
PPI_deg: the number of DEGs in PPI
PPI_cox: the number of cox genes in PPI
deg/PPI: the percentage of DEGs in PPI
cox/PPI: the percentage of cox genes in PPI
cluster_min: the minimum of genes in cluster
cluster_max: the maximum of genes in cluster
LCC.Size: the number of genes in LCC
LCC.seedNum: the number of mutated genes in LCC
LCC_dri: the number of mutated driver genes in LCC
LCC_deg: the number of DEGs in LCC
LCC_cox: the number of COX genes in LCC
deg/LCC: the percentage of DEGs in LCC
cox/LCC: the percentage of cox genes in LCC

PPI_deg	PPI_cox	deg/PPI	cox/PPI	cluster_m	cluster_m	LCC.Size	LCC.seedN	LCC_dri
2784	1937	0.201927	0.138825	22	745	4358	42	96
2785	1937	0.202571	0.1515	7	745	5835	67	112
2787	1938	0.199933	0.149616	10	745	5982	66	115
2782	1937	0.199549	0.158574	10	331	4881	53	100
2787	1939	0.202032	0.133979	17	296	4232	39	87
2786	1939	0.199074	0.152606	9	416	5832	76	110
2785	1938	0.192559	0.148012	4	745	6263	75	127
2788	1937	0.192767	0.139829	5	745	9955	259	154
2788	1939	0.204169	0.154047	2	745	6524	89	130
2785	1937	0.186472	0.150707	12	745	6934	86	123
2788	1938	0.185425	0.13926	4	493	7863	148	139
2787	1939	0.187911	0.158782	12	346	4566	53	89
2788	1939	0.193616	0.165867	2	745	4793	45	95
2786	1937	0.19704	0.143346	2	349	8176	154	136
2784	1939	0.17797	0.148726	7	745	7181	93	127
2786	1939	0.191036	0.148919	13	416	6292	83	113
2786	1938	0.180606	0.135931	7	442	5775	70	107
2787	1936	0.155908	0.144147	8	347	3656	37	72
2787	1939	0.192503	0.142297	3	745	10457	316	154
2787	1938	0.1793	0.141785	27	745	5198	52	97
2785	1938	0.18768	0.1493	19	414	5211	55	100
2785	1938	0.196802	0.148839	7	364	7881	134	127
2787	1938	0.19129	0.145896	30	745	5970	58	112
2787	1937	0.199945	0.138816	6	745	7247	106	135
2784	1937	0.2024	0.149511	17	745	6334	86	122
2788	1939	0.188135	0.15025	26	745	6203	78	119
2786	1938	0.18854	0.151235	20	745	5951	64	117
2784	1937	0.181691	0.148185	39	416	5014	52	90
2787	1938	0.192258	0.160771	29	745	5399	57	106
2787	1938	0.199753	0.151665	2	745	6488	94	120
2784	1937	0.184666	0.130851	54	347	2713	25	68
2789	1938	0.186883	0.146685	14	745	7090	104	135
2786	1938	0.190759	0.142799	6	745	7402	108	131
2788	1938	0.194982	0.145463	14	745	9047	185	148
2783	1935	0.185856	0.148097	11	416	6462	84	129
2783	1936	0.195761	0.139443	17	1095	4812	46	98
2783	1936	0.187786	0.14022	14	745	8073	138	138
2780	1937	0.201295	0.153719	4	347	6642	100	125
2787	1939	0.176159	0.142753	27	745	5478	54	102
2788	1938	0.192212	0.140983	10	745	7242	111	133
2789	1938	0.201089	0.148171	3	745	6425	82	127
2784	1937	0.185714	0.161508	6	745	5040	52	91
2784	1938	0.191349	0.142494	2	455	9825	253	152
2781	1937	0.199379	0.144902	5	745	9013	181	148
2782	1936	0.194834	0.14049	13	745	6698	87	122
2782	1935	0.193588	0.153244	14	745	6519	90	118
2788	1938	0.194148	0.141616	4	1093	10013	280	154
2785	1939	0.178499	0.147449	5	745	6409	90	117
2787	1938	0.187788	0.145015	7	391	6289	83	113
2783	1937	0.192554	0.148922	2	745	5936	68	114

2787	1938	0.190221	0.14222	15	745	6729	90	122
2780	1936	0.180878	0.131288	15	745	5606	66	110
2783	1937	0.198864	0.155303	7	745	5280	48	104
2787	1937	0.184608	0.14477	12	745	5899	74	109
2788	1938	0.185405	0.150354	28	1095	5221	45	103
2784	1939	0.182525	0.153202	10	347	5013	55	97
2788	1938	0.179012	0.145282	26	389	4536	44	90
2782	1938	0.197732	0.143955	15	745	5467	58	112
2787	1938	0.204965	0.143931	27	745	4391	45	91
2787	1939	0.190619	0.136984	4	623	10702	362	161
2787	1938	0.191453	0.137251	2	1093	11162	459	162
2787	1937	0.177879	0.147532	10	365	5470	68	114
2787	1936	0.184569	0.145339	14	745	6908	83	121
2788	1939	0.183403	0.144547	16	745	6254	86	114
2782	1937	0.179473	0.144188	4	745	5583	59	110
2787	1939	0.191703	0.143836	4	745	8461	155	142
2785	1938	0.186875	0.151235	10	430	5303	62	105
2778	1935	0.201447	0.15013	6	745	7327	116	127
2785	1937	0.199965	0.15782	12	347	5671	68	99
2786	1938	0.19267	0.140367	5	477	10095	295	157
2789	1939	0.188743	0.146369	26	745	6183	74	117
2782	1937	0.194739	0.14649	10	745	8021	130	126
2783	1935	0.188238	0.144789	25	431	7618	123	131
2786	1938	0.179385	0.144855	27	745	5792	59	114
2784	1938	0.192703	0.140006	7	745	6414	74	126
2787	1939	0.200045	0.154684	10	307	4409	41	99
2782	1939	0.191527	0.140022	5	441	8970	203	144
2785	1939	0.189935	0.14174	17	396	7034	102	128
2788	1939	0.189591	0.145185	11	745	6936	102	129
2787	1939	0.193541	0.14233	7	745	8670	169	144
2785	1939	0.188025	0.139173	9	416	8802	175	138
2785	1937	0.194584	0.154965	28	351	5982	79	108
2788	1938	0.187011	0.145559	9	416	5235	55	103
2788	1939	0.18541	0.15287	65	745	5593	53	98
2786	1939	0.197288	0.150749	6	745	7005	91	124
2788	1939	0.190069	0.143316	10	745	6545	86	122
2785	1939	0.192818	0.143776	8	349	7463	116	140
2787	1939	0.190707	0.141332	3	745	9276	237	141
2789	1939	0.18979	0.153149	17	745	4858	51	105

LCC_deg	LCC_cox	deg/LCC	cox/LCC
880	605	0.188248	0.130976
1182	884	0.188456	0.131073
1196	895	0.188451	0.131043
974	774	0.188304	0.131109
855	567	0.188311	0.131014
1161	890	0.188307	0.131058
1206	927	0.188494	0.131168
1919	1392	0.188697	0.1311
1332	1005	0.188468	0.131076
1293	1045	0.188622	0.131189
1458	1095	0.188417	0.130972
858	725	0.1884	0.131076
928	795	0.188417	0.13104
1611	1172	0.188371	0.130967
1278	1068	0.188299	0.131146
1202	937	0.188243	0.131014
1043	785	0.188447	0.131088
570	527	0.188413	0.130882
2013	1488	0.188336	0.131031
932	737	0.188438	0.131034
978	778	0.188532	0.131194
1551	1173	0.188405	0.131105
1142	871	0.188464	0.131052
1449	1006	0.188502	0.131011
1282	947	0.188414	0.131091
1167	932	0.188391	0.131022
1122	900	0.188498	0.131123
911	743	0.188516	0.131162
1038	868	0.188732	0.131239
1296	984	0.188464	0.131052
501	355	0.188337	0.131038
1325	1040	0.188599	0.131052
1412	1057	0.188562	0.131168
1764	1316	0.188621	0.131114
1201	957	0.188474	0.131044
942	671	0.188371	0.131041
1516	1132	0.188678	0.131254
1337	1021	0.188334	0.131224
965	782	0.188311	0.131014
1392	1021	0.188442	0.13099
1292	952	0.18851	0.13099
936	814	0.188401	0.131082
1880	1400	0.188363	0.131123
1797	1306	0.188211	0.131091
1305	941	0.18847	0.131156
1262	999	0.188444	0.131071
1944	1418	0.188531	0.131052
1144	945	0.188252	0.131067
1181	912	0.1884	0.131008
1143	884	0.188295	0.131055

1280	957	0.188476	0.131061
1014	736	0.188487	0.131263
1050	820	0.188359	0.1311
1089	854	0.188476	0.130993
968	785	0.188646	0.131132
915	768	0.188388	0.131209
812	659	0.188429	0.130981
1081	787	0.188329	0.131194
900	632	0.188617	0.131159
2040	1466	0.188464	0.13112
2137	1532	0.188413	0.131017
973	807	0.188617	0.131091
1275	1004	0.188527	0.130961
1147	904	0.188378	0.131014
1002	805	0.188317	0.131118
1622	1217	0.188502	0.131146
991	802	0.18852	0.131185
1476	1100	0.188441	0.131258
1134	895	0.188443	0.131064
1945	1417	0.188345	0.131017
1167	905	0.188497	0.131049
1562	1175	0.188368	0.131153
1434	1103	0.188499	0.131062
1039	839	0.188421	0.13107
1236	898	0.188273	0.131061
882	682	0.188464	0.13112
1718	1256	0.188164	0.131146
1336	997	0.188367	0.131146
1315	1007	0.18848	0.131084
1678	1234	0.188425	0.131093
1655	1225	0.188379	0.131155
1164	927	0.188456	0.131073
979	762	0.188608	0.131105
1037	855	0.188493	0.131093
1382	1056	0.188472	0.131173
1244	938	0.188518	0.131111
1439	1073	0.188252	0.131067
1769	1311	0.1884	0.131076
922	744	0.188433	0.131005
