# Supplementary material for: Identification of Warning Transition Points from Hepatitis B to Hepatocellular Carcinoma Based on Mutation Accumulation for the Early Diagnosis and Potential Drug Treatment of HBV-HCC
Source: Oxid Med Cell Longev. 2022 Sep 5;2022:3472179. doi: 10.1155/2022/3472179 (PMC9467738; doi:10.1155/2022/3472179)
Supplement: Supplementary Materials — Supplementary Table 1 and Supplementary Table 2 can be checked in the supplementary files in the submission system. [file 3472179.f1.zip › Supplementary table2-Approved drugs for potential-drug-targets.pdf]

GENE	DRUGBANK-DRUG-NAME	DRUG-GROUP	ACTION	PMID	Diseases
AURKB	DB04703	Hesperidi	approved, investig	30779474、	HepatitisCirrhosisLiver inj
AURKB	DB12010	Fostamati	approved,	inhibitor	28967793、Persistent/chronic adult im
CDK2	DB06616	Bosutinib	approved	inhibitor	29091516	Chronic Granulomatous Leuke
CDK2	DB15442	Trilacicl	approved,	inhibitor	26826116	Reduced risk of chemotherap
HAMP	DB13257	Ferrous s	approved	substrate	26408108	Iron deficiency anemia
RHOB	DB00083	Botulinum	approved,	inhibitor	29230798、	Cervical dystonia
RHOB	DB00083	Botulinum	approved,	inhibitor	15947626、	Urinary incontinence
RHOB	DB00083	Botulinum	approved,	inhibitor	29405250、	Migraine

uryLiver cancer
mune thrombocytopenia
mia
y-induced myelosuppression
